# Clinical Patterns and Outcomes of Eosinophilic Esophagitis in Children and Adolescents at a Tertiary Care Center in Lebanon

**DOI:** 10.3390/children13040513

**Published:** 2026-04-07

**Authors:** Amal Rahi, Rima Hanna-Wakim, Abir Barhoumi, Nadine Yazbeck

**Affiliations:** 1 Department of Pediatrics and Adolescent Medicine, American University of Beirut, Beirut 1107, Lebanon; ar05@aub.edu.lb; 2 Division of Pediatric Infectious Diseases, Department of Pediatrics and Adolescent Medicine, American University of Beirut, Beirut 1107, Lebanon; rh08@aub.edu.lb; 3 Department of Nutrition, American University of Beirut, Beirut 1107, Lebanon; an04@aub.edu.lb; 4 Division of Pediatric Gastroenterology, Department of Pediatrics and Adolescent Medicine, American University of Beirut, Beirut 1107, Lebanon

**Keywords:** eosinophilic esophagitis, children, diet, outcome, retrospective study

## Abstract

**Highlights:**

**What are the main findings?**

- Esophagus can appear grossly normal in EoE despite symptoms and histologic confirmation.
- Resolution of symptoms on dietary elimination and medication is not always correlated with tissue remission.

**What is the implication of the main finding?**

- It is essential to always take biopsies whenever EoE is suspected.
- Frequent endoscopies remain a challenge in pediatric patients, especially in a resource-constrained country.

**Abstract:**

Background: Studies on the clinical presentation of eosinophilic esophagitis and its outcome in children in the Middle East and North African region are scarce. The aim of this 10-year retrospective study was to describe the common clinical manifestations, endoscopic and histological findings, and the response to medication and dietary intervention in children and adolescents with eosinophilic esophagitis. Methods: This study was a retrospective chart review of patients aged 6 months to 18 years who attended the Pediatric Gastroenterology clinic at the American University of Beirut Medical Center between 1 January 2013 and 30 June 2023 and who were diagnosed with eosinophilic esophagitis. Results: A total of 15 patients met the inclusion criteria. The median age at diagnosis was 9 years. Male patients accounted for 73% of our cohort. The most frequent presenting symptoms were dysphagia (80%) and choking (47%). The esophagus appeared normal in 33% of subjects despite histologic confirmation of disease, highlighting the importance of routine biopsies. Adherence to therapy was variable, with 73% of subjects reporting symptom improvement following initial therapy, even in cases where histology remained active. This pattern suggests that symptomatic improvement alone may not reliably reflect disease control and underscores the importance of objective monitoring through follow-up biopsy. Conclusions: The recognition of manifestations of eosinophilic esophagitis in children, early diagnosis, and strict adherence to the diet and medication are essential to prevent long-term complications. In a resource-constrained country like Lebanon, the management remains challenging in view of the burden of dietary restrictions and high cost of procedures and biologics. Socioeconomic feasibility and long-term adherence to diet and medication is as critical as pharmacologic efficacy in determining outcomes in pediatric patients.

## 1. Introduction

Eosinophilic esophagitis (EoE) is a chronic allergen-induced, type 2 immune-mediated disease of the esophagus characterized by symptoms of esophageal dysfunction and an eosinophilic predominant infiltrate in the esophagus [1]. It is characterized by a mixed IgE and non-IgE-mediated reaction to food and/or environmental agents [2]. During the past two decades, EoE incidence and prevalence have risen in many regions worldwide with pooled estimates of about 40 per 100,000 inhabitant-years (0.04%) overall, though this varies widely by region and diagnostic practices [3,4,5]. Increased recognition as well as broader use of endoscopy with biopsies contribute to rising estimates [1,3]. Population-based prevalence of EoE in children is reported to be 19.1 cases per 100.000 children/year [3], with a wide geographic variation from 2.3 in Denmark to 50.5 per 100.000 children in the United States [2]. There are no robust, large-scale epidemiological studies providing definitive prevalence estimates for the entire Middle East region. Available regional data suggest lower reported population prevalence compared with that observed in high-income western countries. This likely reflects underdiagnosis, variable clinical practice, and limited endoscopy/biopsy screening, rather than truly lower disease occurrence [4].

Clinical manifestations of EoE differ by age group as infants and toddlers are more likely to present with refusal to feed or low appetite, vomiting, or failure to thrive. On the other hand, older children present with dysphagia, food impaction and adaptive eating behaviors (food avoidance, prolonged chewing, drinking excessively) [6].

Male predominance and strong association with atopic disease (asthma, allergic rhinitis, atopic dermatitis, food allergy) are reported in most cohorts [7].

Knowledge gaps still exist in the diagnosis and treatment of EoE. These include finding validated noninvasive biomarkers of disease activity, randomized controlled trials comparing dietary intervention to topical steroid or biologic therapy in children. Moreover, there are no conclusive studies addressing criteria for long-term safety and optimal maintenance regimens as well as drivers of progression to stricture and how early treatment alters long-term outcomes [8].

Lebanon represents a meaningful and unique clinical setting for the study of EoE as it combines several epidemiologic, environmental, and dietary characteristics that can provide insights into disease pathogenesis in populations with distinct genetic backgrounds, including higher rates of consanguinity [9]. The traditional Mediterranean diet and regional patterns of allergen exposure may influence immune responses and the development of eosinophilic gastrointestinal disease [10]. Allergic diseases are increasingly recognized in Lebanese children. Epidemiologic studies have reported asthma prevalence ranging from approximately 5 to 8%, while allergic rhinitis affects 25–45% of school-aged children. Food allergy prevalence has been estimated at 4–6% among Lebanese children, with fruits, eggs, nuts, and cow’s milk among the most commonly reported allergens [11,12].

This 10-year retrospective study, carried out in a tertiary care center in Lebanon, aimed to clarify the clinical presentation, endoscopic and histological findings of EoE in children and adolescents, and to evaluate the response to treatment within a lower-middle-income country population.

## 2. Materials and Methods

### 2.1. Study Design

We performed a retrospective chart review of pediatric subjects diagnosed with EoE, followed at the Pediatric Gastroenterology clinic at the American University of Beirut Medical Center (AUBMC) between 1 January 2013 and 30 June 2023. Subjects were identified through medical records using relevant ICD-9 and ICD-10 codes for “eosinophilic esophagitis”, and their charts were subsequently reviewed for data extraction. The study was approved by the Institutional Review Board (IRB) at AUBMC (IRB approval number: BIO-2023-0174).

### 2.2. Inclusion and Exclusion Criteria

The subjects included in the study were patients aged 6 months to 18 years diagnosed with eosinophilic esophagitis based on the following three criteria: symptoms of esophageal dysfunction; at least 15 eosinophils per high power field (eos/hpf) on esophageal biopsy; and an evaluation for non-EoE disorders that cause or potentially contribute to esophageal eosinophilia. Subjects with an unconfirmed diagnosis, or in whom the diagnosis of eosinophilic esophagitis was refuted, and those who were lost to follow-up were excluded from the study.

### 2.3. Data Collection and Study Variables

Subjects who satisfied the inclusion criteria were included in the study and their charts were reviewed. Data extracted from medical records included age, gender, anthropometric measurements, clinical presentation, laboratory results such as serum food immunoglobulin E levels, white blood cell count, eosinophil %, endoscopic findings, and histopathological findings (at presentation, repeated at 6, 12, and 18–24 months), treatment modality, compliance, and outcomes. Food impaction was defined as an event occurring after food ingestion during which solid food is retained in the esophagus.

### 2.4. Upper Endoscopy and Histopathology

All the subjects underwent upper endoscopy. The same senior pediatric gastroenterologist completed all the procedures. An anesthesiologist performed deep sedation. Multiple esophageal biopsies were performed at different levels. Histopathological assessments were performed in the pathology department by a senior pathologist. The number of eosinophils in the most densely involved high power field was counted; each esophageal specimen was also evaluated for basal zone hyperplasia and eosinophil clusters (defined as five or more eosinophils clustered together).

### 2.5. Data Analysis

All statistical analyses were performed using the Statistical Package for the Social Sciences (SPSS), version 23.0 (IBM Corp., Armonk, NY, USA). Descriptive statistics were used to summarize patient demographics and clinical characteristics and are presented as frequencies and percentages. Continuous variables are reported as medians with corresponding ranges or interquartile ranges (IQR), as appropriate.

## 3. Results

We identified 18 pediatric and adolescent patients with EoE followed at our center. During the study period, 652 upper endoscopies were performed making the relative frequency at 2.76%. Three subjects were excluded because they had a single encounter. Eleven subjects (73%) were male. Median age at first presentation was 9 years (IQR 6–13). Symptoms varied with age, with a predominance of loss of appetite and poor weight gain at earlier age, while choking, dysphagia and food impaction mostly present in older subjects. Six subjects were followed up for 6 months, three for one year, and 6 for two years. A total of 38 endoscopies were performed during the study period.

### 3.1. Clinical Presentation

**Figure 1:**
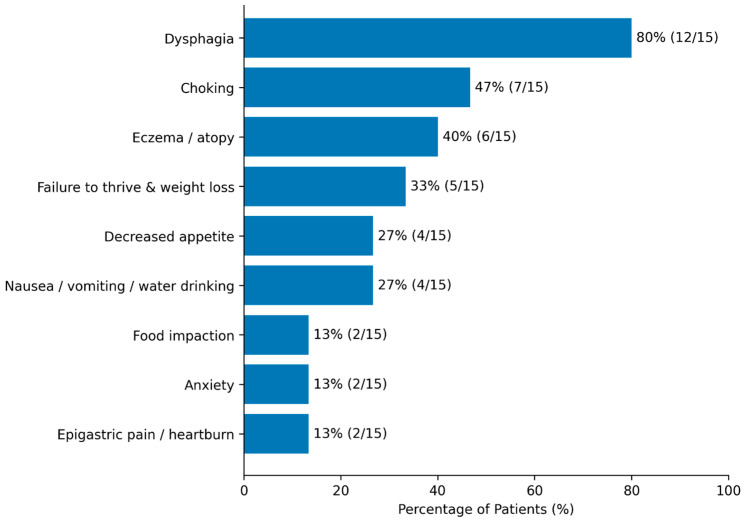
Frequency of symptoms at presentation.

The most frequent presenting symptoms were dysphagia (80%) and choking (47%). There were 2/15 food impaction events requiring endoscopic removal. Feeding refusal or failure to thrive was reported in 33% of subjects. A subset reported ancillary symptoms including heartburn, chest pain, vomiting, or epigastric discomfort. Figure 1 shows the frequency of each of the presenting symptoms.

### 3.2. Atopic Comorbidities

Atopic conditions were identified in 40% of all subjects, including eczema (*n* = 2), allergic rhinitis (*n* = 1), IgE-mediated food allergy (*n* = 7; milk, egg, wheat, kiwi), and family history of EoE (*n* = 1).

### 3.3. Growth Parameters

Five subjects presented with failure to thrive or underweight, while three subjects were overweight. Most subjects maintained stable height and weight z-scores over the course of follow-up, though a subset had impaired linear growth.

### 3.4. Laboratory Findings

Peripheral blood eosinophilia was present in most subjects, ranging from 6 to 10% of the white blood cell count in typical cases. Specific IgE to cow’s milk was positive in 5 subjects, ranging from low-positive (class 1) to high (class 3).

### 3.5. Endoscopic Findings and Histology

Initial endoscopy demonstrated classic EoE features in the majority of subjects: linear furrows (7/15), trachealization/rings (2/15), and food impaction requiring intervention (2/15). In several cases (5/15), the esophagus appeared grossly normal despite histologic confirmation of disease, highlighting the importance of routine biopsies.

At diagnosis, all subjects met histologic criteria for EoE with peak intraepithelial eosinophil counts ≥15 eos/hpf. Median peak eosinophil count was 60 eos/hpf (range 20–100). Several subjects demonstrated basal cell hyperplasia, spongiosis, and eosinophilic micro abscesses, consistent with active disease. Table 1 details the endoscopic and histologic findings upon presentation.

### 3.6. Treatment Strategies

All subjects were initiated on proton pump inhibitor (PPI) therapy at diagnosis at a dose of 2 mg/kg once a day or 1 mg/kg per dose twice daily, combined with swallowed topical corticosteroids (fluticasone propionate or budesonide) in 14/15 cases. Dietary elimination therapy was recommended for all subjects, most commonly with dairy elimination (87%), while the remaining subjects were advised to follow a broader elimination diet that excluded soy, wheat and eggs. The same experienced dietitian gave the recommended dietary modification. Biologics (dupilumab) were offered to one subject with refractory disease, but it was not initiated due to financial limitations.

### 3.7. Compliance

Adherence to therapy was variable. Seven subjects (47%) demonstrated good compliance with both medications and dietary restrictions, resulting in symptom control and histologic remission at 6–12 months. Five subjects (33%) had inconsistent compliance (skipping medications, partial diet adherence), leading to persistent or recurrent esophageal eosinophilia. Three subjects (20%) were lost to follow-up within two years of diagnosis due to socioeconomic or personal reasons.

### 3.8. Clinical Course

**Figure 2:**
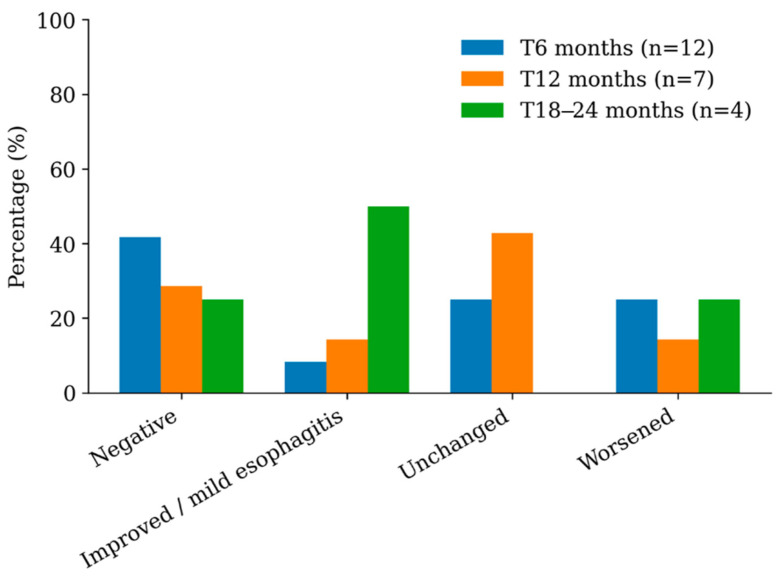
Esophageal pathology results at 6, 12 and 18–24 months.

Symptom improvement was documented in 11/15 subjects (73%) following initial therapy, even in cases where histology remained active. This pattern suggests that symptomatic improvement alone may not reliably reflect histologic disease control in EoE and underscores the importance of objective monitoring through follow-up endoscopy and biopsy. Repeat endoscopy was performed at 6 months for 12 subjects; at 12 months, for seven subjects, and at 18–24 months for 4 subjects. Histologic remission (negative biopsies) was achieved in 42% of subjects at 6 months, typically in those who adhered to diet and medications. Histologic persistence (≥15 eos/hpf) and worsening, with some counts exceeding 80–100 eos/hpf, were observed despite partial symptom control in 50% of subjects. These results highlight the varied nature of EoE treatment outcomes and further emphasize that clinical symptoms may not align with underlying tissue inflammation. Relapse after initial remission occurred in three subjects, associated with reintroduction of dairy or discontinuation of medications, indicating that consistent long-term treatment and dietary restriction are vital for disease control. Figure 2 shows pathology findings at 6, 12 and 18–24 months.

### 3.9. Long-Term Follow-Up

The median follow-up duration was 3 years (range: 6 months to 9 years). Sustained remission off therapy was rare; only two subjects achieved repeated negative biopsies and remained asymptomatic while off both dietary modification and medications. The majority of subjects required ongoing therapy to maintain disease control, highlighting the chronic and relapsing nature of pediatric eosinophilic esophagitis.

## 4. Discussion

To the best of our knowledge, this study is the first reporting on the clinical, endoscopic, and histologic characteristics of pediatric patients with EoE in Lebanon. Pediatric gastrointestinal endoscopy is available in only a limited number of tertiary centers (approximately 5–7 nationwide), mostly concentrated in Beirut, with a small specialized workforce, underscoring potential disparities in access to care in the absence of a centralized registry.

Our retrospective analysis provides valuable insights on the disease relative frequency, presentation, diagnostic evaluation, treatment approach and outcomes among Lebanese children and adolescents with EoE. In a recent systematic review published in 2026, the authors examined the epidemiology of EoE in the Middle East, analyzing 67 studies that included a total of 2870 pediatric and adult patients diagnosed across 11 countries. The majority of the data originated from Saudi Arabia, Iran, Egypt, and Turkey, with only two case reports and a single abstract describing adult patients with EoE from Lebanon [13].

Eosinophilic esophagitis predominantly affects children and young adults of the male sex [6]. In our cohort, a high predominance of males was observed (73%), and the average age at diagnosis was 9 years (IQR 6–13), similar to data from an Italian cohort of 56 patients [14]. The most common presenting symptom was dysphagia, similar to other studies in the region [13] and worldwide [2].

The clinical manifestations of EoE are age dependent. Although feeding issues, namely vomiting, are prevalent in the younger age groups, dysphagia and food impaction are the presenting symptoms in older children and adolescents [1,15]. As described by Dellon et al. in 2018, there is significant phenotypic variability in the presentation of EoE based on age and duration of the disease [16]. In our cohort, decreased appetite, weight loss, nausea, and vomiting occurred mostly in patients less than 9 years of age, while dysphagia and choking were significantly present in those 10 years and older. Food impaction was the presenting symptom in two adolescent subjects. Recognizing these symptoms is crucial for the early identification of EoE as they may be subtle. In addition, some coping behavioral mechanism can mask the signs, thereby further delaying the diagnosis. These coping mechanisms include eating slowly, excessive chewing, taking small bites, drinking liquids to facilitate swallowing, and preferentially selecting softer foods while avoiding harder textures [16].

In our cohort, 40% of the subjects had an associated atopic condition. Similar studies conducted in Saudi Arabia and Jordan reported a prevalence of atopic diseases ranging from 33 to 47% among pediatric patients with EoE [17,18]. Furthermore, a systematic review of EoE in Middle Eastern countries demonstrated a strong association between atopic disorders and EoE, supporting the concept that pediatric EoE in this region frequently occurs within the context of broader allergic or immunological dysregulation [13].

Although endoscopic findings can be very suggestive of EoE like ringed appearance or linear furrows with white exudates, normal endoscopic appearance does not rule out EoE. In this study, furrowing (present in 47% of subjects) was the most common endoscopic finding, while 33% of subjects had normal-appearing esophagus, proving the necessity to always take biopsies. These results were similar to studies from Saudi Arabia and Jordan, where linear furrows and loss of vascular markings were the most prevalent features, but only 5.4 and 9.5%, respectively, had a normal esophagus as compared to 33% in our cohort [18,19]. The American College of Gastroenterology recommends obtaining a minimum of six biopsies from at least two esophageal levels to accurately assess treatment response and monitor disease progression [1]. To enhance diagnostic sensitivity, we obtained esophageal samples from both proximal and distal esophagus. Basal cell hyperplasia, spongiosis, and eosinophilic degranulation were the most frequently observed histopathological features, consistent with findings reported in the previous studies [19,20].

Therapy for EoE aims to achieve clinical and histological remission, prevent esophageal fibrosis, and improve quality of life. It is of utmost importance to have close follow-up and repeated gastroscopies and biopsies since clinical improvement alone is not sufficient and needs to be correlated to tissue remission [21]. Although most of the subjects (73%) in our cohort had resolution of symptoms after initial therapy, 12 of them repeated gastroscopy at 6 months, and only 42% had negative biopsies, whereas 50% had persistent or worsening findings.

Several studies have demonstrated a discordance between clinical symptoms and histologic disease activity in eosinophilic esophagitis [22]. Patients may report symptomatic improvement despite persistent eosinophilic inflammation, while others remain symptomatic despite histologic remission. These findings highlight the limitations of symptom-based monitoring and support the need for objective histologic assessment through follow-up endoscopy and biopsy [23,24].

Endoscopic surveillance remains essential in the management of EoE; however, frequent endoscopies pose an important challenge in pediatric patients since they often require anesthesia and hospitalization [21].

In pediatrics, the cornerstones of therapy are proton pump inhibitors, topical steroids, and food elimination diets [25]. Adherence to long-term pharmacologic therapy or dietary elimination strategies can be challenging, particularly in children and adolescents, and persistent nonadherence has been associated with ongoing esophageal inflammation and progression to fibrostenotic disease [16]. Empiric elimination diets are currently the most practical and widely supported dietary strategy for EoE, as they remove food groups known to commonly trigger the disease without relying on allergy testing [21]. In a prospective observational outcome study by Kagalwalla et al., including 78 pediatric patients with EoE from 4 centers in the US, cow’s milk was the most common food trigger identified in 85% of patients [26]. All our patients were maintained on a dairy and soy free Mediterranean diet. Mediterranean diet is a dietary pattern that inherently emphasizes anti-inflammatory foods focusing on fruits, vegetables, whole grains, legumes, nuts, extra virgin olive oil, and fish, which are rich in antioxidants and omega-3 fatty acids that may help reduce esophageal inflammation. The Mediterranean diet principles align with broad-spectrum, plant-forward foods using mainly fresh produce by prioritizing local and seasonal food items. This automatically reduces reliance on processed item [10]. In the Lebanese context, adherence to a Mediterranean-style dietary pattern is largely feasible due to its close alignment with traditional dietary practices. However, elimination of dairy products represents a significant challenge, as dairy is deeply embedded in local culinary habits and contributes to protein and calcium needs. Apart from dairy restriction, the remaining components of the Mediterranean diet are generally well accepted and relatively easy to implement and sustain over time.

Dupilumab, is a fully human monoclonal antibody directed against the IL-4 receptor alpha component that inhibits the signaling of both IL-4 and IL-13, which are key initiators of type 2 inflammation. It is approved for the treatment of atopic dermatitis, asthma, and eosinophilic esophagitis [1,27]. A retrospective multicenter study conducted across three Saudi tertiary centers by Hasosah et al. evaluated children with confirmed eosinophilic esophagitis and demonstrated that dupilumab, used as second-line therapy, was effective in refractory cases, achieving symptom relief in 82% of patients, endoscopic improvement in 73%, and histologic remission in 90% [28]. In Lebanon, dupilumab is not yet approved by the Ministry of Public Health for the treatment of EoE, and the price is prohibitive for most patients.

It is important to acknowledge the limitations of this study. First, its retrospective design that can lead to the potential for information and selection bias. Second, being a single-center study may hinder generalizability of the findings to a wider population. Finally, the small sample size may reduce the ability to draw definitive conclusions. These factors underscore the need for larger, prospective, multicenter studies to further characterize the clinical and epidemiological features of pediatric EoE in the region.

## 5. Conclusions

This study provides valuable insights into the clinical presentation, assessment, therapeutic strategies, and outcomes of pediatric EoE. Our findings reinforce the need for early recognition, timely histologic confirmation, and structured longitudinal follow-up to optimize disease control in this chronic immune-mediated condition. In particular, in low-middle-income countries such as Lebanon, socioeconomic feasibility and long-term adherence to diet and medication may be as critical as pharmacologic efficacy in determining outcomes in pediatric patients with EoE. This study highlights the challenges of real-world management, particularly those related to dietary adherence in children and adolescents, and the availability of costly new therapies like biologics. These practical barriers should be carefully considered when individualizing treatment strategies and designing future interventional studies aimed at improving long-term outcomes.

## Figures and Tables

**Table 1:** Endoscopic and histopathological features at presentation (*N* = 15).

Finding	*N* (%)
**Endoscopic findings**	
Furrowing	7 (47%)
Normal esophagus	5 (33%)
Loss of vascular markings	2 (13%)
Ring formation	2 (13%)
Ulcer	2 (13%)
Whitish exudates	1 (7%)
Stricture	0 (0%)
**Histopathological findings**	
Median eosinophils/hpf	60 (range 20–100)
Basal cell hyperplasia	5 (33%)
Spongiosis	3 (20%)
Eosinophilic degranulation	2 (13%)
Eosinophilic microabscess	1 (7%)

## Data Availability

The original contributions presented in this study are included in the article. Further inquiries can be directed to the corresponding author.
